# Correction to: SpectralTAD: an R package for defining a hierarchy of topologically associated domains using spectral clustering

**DOI:** 10.1186/s12859-020-03710-3

**Published:** 2020-08-27

**Authors:** Kellen G. Cresswell, John C. Stansfield, Mikhail G. Dozmorov

**Affiliations:** Department of Biostatistics, Virginia Commonwealth University, Richmond, VA USA

**Correction to: BMC Bioinformatics 21, 319 (2020)**

**https://doi.org/10.1186/s12859-020-03652-w**

Following publication of the original article [1], the authors identified misformatted equations in the published article. The correctly formatted equations are given below.

1. Calculating the normalized symmetric Laplacian:

$$ \overline{L}={D}^{-\frac{1}{2}}C{D}^{-\frac{1}{2}} $$

2. Solve the generalized eigenvalue problem:

$$ \overline{L}\overline{V}=\lambda \overline{V} $$

3. The result is a matrix of eigenvectors ${\overline{V}}_{w\times k}$, where *w* is the window size, and *k* is the number of eigenvectors used, and a vector of eigenvalues where each entry *λ*_*i*_ corresponds to the *i*_*th*_ eigenvalue of the normalized Laplacian $\overline{L}$.

4. Normalize rows and columns to sum to 1:

$$ \hat{V_{i.}}=\frac{\overline{V_{i.}}}{\left\Vert {\overline{V}}_{i.}\right\Vert } $$

5. Find the mean silhouette score over all possible numbers of clusters *m* and organize into a vector of means:

$$ {\overline{s}}_m=\frac{\sum_{i=1}^m{s}_i}{m} $$

6. Find the value of *m* which maximizes ${\overline{s}}_m$

The original article has been updated.
