# Supplementary material for: SpectralTAD: an R package for defining a hierarchy of topologically associated domains using spectral clustering
Source: BMC Bioinformatics. 2020 Jul 20;21:319. doi: 10.1186/s12859-020-03652-w (PMC7372752; doi:10.1186/s12859-020-03652-w)
Supplement: Supplementary file 1 — Additional file 1: Supplementary Material. An overview of previous methods, parameter selection, methods, and results not included in the main manuscript. [file 12859_2020_3652_MOESM1_ESM.pdf]

Supplementary Material

Previous Methods

Many of the first and most popular TAD detection methods were based on the directionality index, which is a function of the average upstream and downstream interactions. This metric was then used as a parameter in a hidden Markov model to establish the location of TAD boundaries [1]. The basis of this method was the fact that boundary regions are expected to interact with downstream regions more than upstream regions. This method was followed by a number of methods designed to calculate non-hierarchical TADs such as `Armatus` [2], `HiCseg` [3], `TADLib` [4], `TopDom` [5], `Arrowhead` [6], `TADbit` [7] and `RHiCDB` [8]. Another intuitive metric, the insulation index [9], uses a sliding window approach to sum up contacts within a given region surrounding each locus. As TADs are regions of increased contacts, they can easily be identified via a contact count cutoff. Some tools, such as the `TADtool` Python package [10], implement both metrics to call TADs. `HiCDB` uses an extension of the conventional insulation index that corrects for background noise.

The detection of hierarchical TAD structures was first introduced by Fraser J. et al. [11] who used single-linkage clustering to create a hierarchical structure of meta-TADs which contain smaller sub-TADs. Since this discovery, there has been a lack of publicly available, user-friendly, hierarchical TAD callers. A hierarchical TAD caller refers to a tool that finds TADs and sub-TADs contained within them. To date, the choice of hierarchical TAD callers remains limited.

The first publicly available tool for hierarchical TAD calling, `TADtree` [12] worked by creating TAD "forests" containing hierarchical "trees" of TADs. `TADtool` similarly provides hierarchical TAD detection and visualization [10]. Other hierarchical TAD finders include `HiTAD` [13] and `IC-Finder` [14] which take dynamic programming, hidden Markov model-dynamic programming hybrid, and probabilistic approach, respectively. `ClusterTAD` [15] introduced a traditional hierarchical clustering-based approach to TAD classification. Another method, `rGMAP` [16] has arisen as a potentially useful tool for TAD detection. This method utilizes a Gaussian Mixture Model and a z-test of proportions to identify TADs. The model is then run iteratively to partition TADs into sub-TADs, but in practice is limited to two levels of TADs. `CaTCH` [17] is another approach that uses a novel measure called reciprocal insulation (RI) and iteratively partitions TADs into a hierarchy based on different thresholds of this value. More recently, a method called `OnTAD` was proposed [18]. This method uses `TopDom`, a single-level TAD caller that uses a statistical test on upstream and downstream contacts, to find all possible TAD boundaries and then to select a final configuration using a dynamic programming algorithm [5]. Of these methods, `TADtool`, `TADtree` and `TADLib` are Python-based. `IC-Finder` and `ClusterTAD` are MATLAB based with `ClusterTAD` also including Java implementation while `rGMAP` and `CaTCH` are available as R packages. Although some comparison of TAD detection tools has been performed [19–21], this diversity leaves the choice of the most appropriate method uncertain.

Hi-C data, represented as an adjacency matrix, naturally lends itself to the use of graph theory [22–24]. `Arboretum-HiC` first introduced the idea of using Laplacian-graph segmentation to find structures in Hi-C data [25]. This method used a spectral clustering approach too simultaneously find 3D structures between multiple matrices. Chen et al. [26] proposed a method that framed the contact matrix as a weighted adjacency matrix and used recursive partitioning of the Fiedler vector to identify TADs. `MrTADFinder` [27] and `HiTAD` [13] take a similar approach but address the question as a community detection problem. Most recently, `3DNetMod` was introduced, which treats

the Hi-C matrix as a network and uses network modularity to cluster the TADs [28]. This method is also designed to find hierarchies of TADs. Network theoretical, or more broadly graph-theoretical, approaches have a promise to provide us with a data-driven method of identifying TADs that takes the entire structure of loci-loci interactions into account.

R/Bioconductor is the de facto gold standard programming language for the genomics community [29]. Currently, the number of TAD callers implemented in the R programming language are limited and include `HiCseg` [3], `TopDom` [5], `rGMAP`[16], `CaTCH` [17] and `HiCDB` [8]. `HiCseg` is the only TAD-calling specific R package available on CRAN or Bioconductor, while `TopDom` is a downloadable R script. `HiCDB`, `rGMAP` and `CaTCH` are available on GitHub. Another tool, `RobustTad`, is in development and currently only provides a metric for TAD calling. It will potentially provide a new option for R users [30]. Additionally, the `HiTC` R package [31] has functionality for calculating the directionality index but does not provide any tools for TAD identification. Neither `TopDom` nor `HiTC` can operate on the commonly used $n \times n$ contact matrices in text format. `TopDom` requires the data to be formatted as an $n \times n + 3$ matrix with the first three columns corresponding to the genomic coordinates. `HiTC` requires the user to transform the data into their package-specific `HTCexp` object. `HiCseg` can be used to analyze $n \times n$ text matrices but forces users to assume a distribution of contacts and estimate the number of TADs before running. These factors aren't always clearly apparent from the data and thus require constant tweaking to account for different levels of noise, sparsity, and resolution of Hi-C data. Although `HiCDB` can process $n \times n$ contact matrices, it requires matrices from multiple chromosomes, thus limiting the analysis of single-chromosome data. Additionally, it requires data to have a resolution of 5kb, 10kb, or 40kb. The aforementioned limitations limit the choice of R-based tools for direct comparison.

Identification and removal of gaps

We define gaps as regions where there is no coherent connectivity structure. There are two types of gaps, those with no contacts at all (centromeres and unsequenced regions), and regions where contacts exist but TADs are not present. The first category of gaps is removed by simply getting rid of loci with more than 95% zero contacts. Since there are situations when TADs span unsequenced regions, we allow TADs to start on one side of a gap and end on another. This is done by essentially treating regions on either side of a gap as being adjacent. The second category of gaps is detected using the silhouette score. Regions where contacts are present but no TADs exist will frequently have low silhouette scores due to the poor similarity between each locus within the region. As a result, we can detect this type of gap by treating it as a potential TAD then filter it out if its silhouette score is lower than .25.

Simulating levels of noise, sparsity, and sequencing depth

Simulated contact matrices [20] (Supplementary Table S4) were modified to simulate various levels of noise, sparsity, and sequencing depth. The noise was added by randomly selecting a percentage (4%, 8%, 12%, 16%, and 20%) of entries in the matrix and adding a constant of two to these entries. Entries were sampled with replacement meaning certain entries may have received more noise than others. In summary, we used five replicates at each noise level, totaling 25 simulated matrices.

We created two extra sets of contact matrices for simulating sparsity and sequencing depth. To mimic sparsity, we took the five simulated matrices with the minimum level of noise (4%) and introduced 90%, 75%, 50%, 25%, or 10% of zeros uniformly at random, totaling 25 matrices. To

simulate changes in sequencing depth, we took the same five minimum noise matrices and applied the downsampling procedure adapted from [32]. Briefly, the full contact matrix was converted into a vector of pairwise individual intra-chromosomal contact counts. The vector was downsampled uniformly at random proportional to the level of downsampling (1/2, 1/4, 1/8, and 1/16). Following downsampling, the vector was re-binned to the original contact matrix. This procedure produced a set of 20 matrices (five matrices, each modified by four levels of downsampling) with varying levels of downsampling.

Choosing TAD caller parameters

HiCseg

Following the suggestions in the `HiCseg` manuscript [3] regarding unnormalized data, we use a Poisson distribution. To allow for the detection of all possible changepoints, we set the maximum number of changes (TADs) to be equal to the rows of the contact matrix divided by 3. This is in line with the optimal parameters laid out in [20]. Since we are using traditional contact matrices, a block diagonal model is used.

TopDom

For all analysis between 10kb and 50kb, including simulations, we use the suggested window size of 5 [5]. As we get into more high resolution, a window size of 5 and a window size of 20 was tested. The best results from the two are reported. The reasoning behind the large window size is to account for the fact that the optimal window size was never determined for high-resolution data. By choosing the maximum window size of 20, we are allowing for more bins to be included in a TAD, giving more realistic results.

OnTAD

We set parameters such that the minimum TAD size is 5 bins, and the maximum is equal to 2mb/resolution. These parameters are in line with those used for `SpectralTAD`. Additionally, we use a penalty term of .1 to filter out weak TADS. This was chosen according to the suggested penalty listed in the `OnTAD` vignette (https://github.com/anlin00007/OnTAD).

rGMAP

For `rGMAP`, we use the suggested default parameters with resolution set to the resolution of the data. Maximum distance is set to 2mb with a minimum window size of 25 bins and a maximum of 100 bins.

Normalization of Hi-C data

Normalization of Hi-C data matrices is a common step in Hi-C data analysis [33–39]. To test for the effect of normalization on the detection of TADs, we applied iterative correction and eigenvector decomposition (ICE) [34], Knight-Ruiz (KR) [35, 40] normalization, and the Square Root Vanilla

Coverage (sqrtVC) [40] to the simulated and experimental Hi-C matrices. ICE was implemented using the `ICE` function from the `dryHiC` R package version 0.0.0.9000. KR-normalization [41] was performed using Juicer [6], and sqrtVC normalization was performed using a function written by the authors.

Measuring association of TAD boundaries with genomic annotations

To test for the enrichment of a genomic annotation at a given TAD boundary, we measure the total number of annotations within 50kb of the boundary point on either side. The flanking region accounts for the fact that a TAD boundary is a single point. Flanking also helps to correct for impreciseness due to issues like overlapping TAD boundaries and differences in resolutions. We specifically choose 50kb for comparison of genomic features because it allows for at least one bin of wiggle room for all resolutions used in this paper. By keeping the size of the flanking region consistent across resolutions, we can directly compare enrichment at TAD boundaries across resolutions.

A permutation test was used to quantify the enrichment of overlap of TAD boundaries with genomic annotations. Briefly, the difference in the mean number of genomic annotations within 50kb of each boundary and that of for all other regions was calculated (observed enrichment). Two sets of bins, one the size of the TAD boundaries and another the size of all other regions were sampled without replacement, and the difference in the mean number of genomic annotations in the corresponding sets was calculated (expected enrichment). This procedure was repeated 10000 times. We determined the permutation p-value by taking the number of expected mean differences that were greater than the observed difference in means between TAD boundaries and all other regions of the chromosome, and dividing by 10000. $\alpha = 0.05$ was set to assess statistical significance. For all hierarchical TAD callers, only the first level of TAD boundaries was used.

Jaccard and its modified version as a measure of similarity between TADs

Traditionally, Jaccard is used as a measure of overlap between sets. We use it as a measure of overlap between TAD boundaries. Given a set of TAD boundaries A and B, we define the Jaccard as:

$$J = \frac{A \cap B}{A \cup B}$$

In plain terms, this is the set of shared boundaries divided by the total number of unique boundaries.

While we expect TADs called at different resolutions of the same Hi-C data to be nearly identical, TADs called from a higher-resolution data may be finer partitioned than those called from lower-resolution data. The traditional Jaccard measure will penalize for these finer TADs even though the original boundaries were detected. We introduce a modified Jaccard score of J_a, which accounts for the difference between TADs detected across resolutions.

$$J_a = \frac{A \cap B}{min(|A|, |B|)}$$

Here, A and B are two sets of TAD boundaries and $|A|$ and $|B|$ indicates the size of sets (Supplementary Figure S1). This method is identical to the Jaccard statistic, but instead of dividing by the union of A and B we divide by the smallest size. A score of 1 indicates that all of set A is contained in a subset of B or vice-versa. This is in contrast to traditional Jaccard where a score of 1 indicates that all boundaries in A and B are identical.

Modified Jaccard with a flank

The modified Jaccard can be extended to account for the impreciseness of TAD callers or differences in the resolution which make finding identical TADs impossible. For instance, half of the loci in a 25kb resolution contact matrix aren't actually in the 50kb resolution version of the same matrix but in one of the neighboring bins. This "off-by-one" error is accounted for by extending TAD boundary points at higher resolution by flanking regions of size f. Consequently, the modified Jaccard formula becomes:

$$J_a = \frac{\mathbf{A} \cap \mathbf{B}}{min(|A|, |B|)}$$

where $\mathbf{A} = \{A, A + f, A - f\}$ and $\mathbf{B} = \{B, B + f, B - f\}$.

When comparing the 50kb and 25kb resolution matrices, we can set $f = 25000$ to make up for any difference in resolution. Note that modified Jaccard is used only when comparing boundaries between different resolutions; otherwise, the traditional Jaccard statistic is used.

Runtime analysis

One general drawback of spectral clustering is the fact that it scales poorly to large matrices. Traditionally, the three main bottlenecks are 1) the creation of the distance matrix, 2) the creation of the Laplacian matrix, and 3) the eigenvalue decomposition. Letting n equal the number of loci in the genome, the distance matrix creation has a complexity of $\mathrm{O}(n^3)$. The Laplacian matrix creation involves two matrix multiplication steps. The traditional multiplication step costs $\mathrm{O}(2n^3)$, and the eigendecomposition step costs $\mathrm{O}(n^3)$. In total, these bottlenecks result in computational complexity of $\mathrm{O}(4n^3)$ or more simply $\mathrm{O}(n^3)$, cubic complexity.

Our method manages to avoid all of these bottlenecks. The first bottleneck is avoided because the contact matrix is itself an adjacency matrix and doesn't require transformation to a distance matrix. The second bottleneck is solved by calculating relatively small Laplacian matrices for each window instead of calculating the Laplacian matrix of the entire contact map. The total number of windows for a given chromosome of size n with window size w is equal to $\sim \frac{n}{w}$ with some discrepancy for rounding and variable TAD size. Accordingly, the computational complexity of Laplacian matrix creation over a given number of windows is equal to $O(w^3 \frac{n}{w})$ or more simply $O(n)$. The third bottleneck is similarly addressed by the windowed approach. The window makes the computation complexity of the eigendecomposition equal to $O(Kw^2nm)$, where K is the number of eigenvalues and m is the number of iterations for convergence of the eigensolver. This reduces to $O(n)$. As a result of these steps, the windowed spectral clustering algorithm reduces to the computational complexity of $O(2n)$, or simply linear complexity $O(n)$.

References

1. Dixon JR, Selvaraj S, Yue F, Kim A, Li Y, Shen Y, et al. Topological domains in mammalian genomes identified by analysis of chromatin interactions. Nature. 2012;485:376–80.

2. Filippova D, Patro R, Duggal G, Kingsford C. Identification of alternative topological domains in chromatin. Algorithms for Molecular Biology. 2014;9:14.

3. Levy-Leduc C, Delattre M, Mary-Huard T, Robin S. Two-dimensional segmentation for analyzing hi-c data. Bioinformatics. 2014;30:i386–92.

4. Wang X-T, Dong P-F, Zhang H-Y, Peng C. Structural heterogeneity and functional diversity of topologically associating domains in mammalian genomes. Nucleic Acids Research. 2015;43:7237–46.

5. Shin H, Shi Y, Dai C, Tjong H, Gong K, Alber F, et al. TopDom: An efficient and deterministic method for identifying topological domains in genomes. Nucleic Acids Research. 2016;44:e70–0.

6. Durand NC, Shamim MS, Machol I, Rao SSP, Huntley MH, Lander ES, et al. Juicer provides a one-click system for analyzing loop-resolution hi-c experiments. Cell Systems. 2016;3:95–8.

7. Serra F, Baù D, Goodstadt M, Castillo D, Filion GJ, Marti-Renom MA. Automatic analysis and 3D-modelling of hi-c data using tadbit reveals structural features of the fly chromatin colors. PLoS Comput Biol. 2017;13:e1005665.

8. Chen F, Li G, Zhang MQ, Chen Y. HiCDB: A sensitive and robust method for detecting contact domain boundaries. Nucleic Acids Research. 2018.

9. Crane E, Bian Q, McCord RP, Lajoie BR, Wheeler BS, Ralston EJ, et al. Condensin-driven remodelling of x chromosome topology during dosage compensation. Nature. 2015;523:240–4.

10. Kruse K, Hug CB, Hernandez-Rodriguez B, Vaquerizas JM. TADtool: Visual parameter identification for tad-calling algorithms. Bioinformatics. 2016;32:3190–2.

11. Fraser J, Ferrai C, Chiariello AM, Schueler M, Rito T, Laudanno G, et al. Hierarchical folding and reorganization of chromosomes are linked to transcriptional changes in cellular differentiation. Mol Syst Biol. 2015;11:852.

12. Weinreb C, Raphael BJ. Identification of hierarchical chromatin domains. Bioinformatics. 2016;32:1601–9.

13. Wang X-T, Cui W, Peng C. HiTAD: Detecting the structural and functional hierarchies of topologically associating domains from chromatin interactions. Nucleic Acids Res. 2017;45:e163.

14. Haddad N, Vaillant C, Jost D. IC-finder: Inferring robustly the hierarchical organization of chromatin folding. Nucleic Acids Res. 2017;45:e81.

15. Oluwadare O, Cheng J. ClusterTAD: An unsupervised machine learning approach to detecting topologically associated domains of chromosomes from hi-c data. BMC Bioinformatics. 2017;18:480.

16. Yu W, He B, Tan K. Identifying topologically associating domains and subdomains by gaussian mixture model and proportion test. Nature Communications. 2017;8.

17. Zhan Y, Mariani L, Barozzi I, Schulz EG, Blüthgen N, Stadler M, et al. Reciprocal insulation analysis of hi-c data shows that TADs represent a functionally but not structurally privileged scale in the hierarchical folding of chromosomes. Genome Research. 2017;27:479–90.

18. An L, Yang T, Yang J, Nuebler J, Li Q, Zhang Y. Hierarchical domain structure reveals the divergence of activity among TADs and boundaries. 2018.

19. Ay F, Noble WS. Analysis methods for studying the 3D architecture of the genome. Genome Biol. 2015;16:183.

20. Forcato M, Nicoletti C, Pal K, Livi CM, Ferrari F, Bicciato S. Comparison of computational methods for hi-c data analysis. Nat Methods. 2017;14:679–85.

21. Nicoletti C, Forcato M, Bicciato S. Computational methods for analyzing genome-wide chromosome conformation capture data. Curr Opin Biotechnol. 2018;54:98–105.

22. Boulos RE, Arneodo A, Jensen P, Audit B. Revealing long-range interconnected hubs in human chromatin interaction data using graph theory. Phys Rev Lett. 2013;111:118102.

23. Wang Y, Sarkar P, Ursu O, Kundaje A, Bickel PJ. Network modelling of topological domains using hi-c data. arXiv preprint arXiv:170709587. 2017.

24. Wang H, Duggal G, Patro R, Girvan M, Hannenhalli S, Kingsford C. Topological properties of chromosome conformation graphs reflect spatial proximities within chromatin. In: Proceedings of the international conference on bioinformatics, computational biology and biomedical informatics. New York, NY, USA: ACM; 2013. pp. 306:306–15.

25. Fotuhi Siahpirani A, Ay F, Roy S. A multi-task graph-clustering approach for chromosome conformation capture data sets identifies conserved modules of chromosomal interactions. Genome Biol. 2016;17:114.

26. Chen J, Hero AO 3rd, Rajapakse I. Spectral identification of topological domains. Bioinformatics. 2016;32:2151–8.

27. Yan K-K, Lou S, Gerstein M. MrTADFinder: A network modularity based approach to identify topologically associating domains in multiple resolutions. PLOS Computational Biology. 2017;13:e1005647.

28. Norton HK, Emerson DJ, Huang H, Kim J, Titus KR, Gu S, et al. Detecting hierarchical genome folding with network modularity. Nature Methods. 2018;15:119–22.

29. Gentleman R, Carey V, Huber W, Irizarry R, Dudoit S. Bioinformatics and computational biology solutions using r and bioconductor. Springer Science & Business Media; 2006.

30. Dali R, Bourque G, Blanchette M. RobusTAD: A tool for robust annotation of topologically associating domain boundaries. bioRxiv. 2018. doi:10.1101/293175.

31. Servant N, Lajoie BR, Nora EP, Giorgetti L, Chen C-J, Heard E, et al. HiTC: Exploration of high-throughput 'c' experiments. Bioinformatics. 2012;28:2843–4.

32. Yardımcı GG, Ozadam H, Sauria MEG, Ursu O, Yan K-K, Yang T, et al. Measuring the reproducibility and quality of hi-c data. 2017.

33. Lieberman-Aiden E, Berkum NL van, Williams L, Imakaev M, Ragoczy T, Telling A, et al. Comprehensive mapping of long-range interactions reveals folding principles of the human genome. Science. 2009;326:289–93.

34. Imakaev M, Fudenberg G, McCord RP, Naumova N, Goloborodko A, Lajoie BR, et al. Iterative correction of hi-c data reveals hallmarks of chromosome organization. Nat Methods. 2012;9:999–1003.

35. Knight PA, Ruiz D. A fast algorithm for matrix balancing. IMA Journal of Numerical Analysis. 2012;33:1029–47.

36. Cournac A, Marie-Nelly H, Marbouty M, Koszul R, Mozziconacci J. Normalization of a chromosomal contact map. BMC Genomics. 2012;13:436.

37. Hu M, Deng K, Selvaraj S, Qin Z, Ren B, Liu JS. HiCNorm: Removing biases in hi-c data via poisson regression. Bioinformatics. 2012;28:3131–3.

38. Li W, Gong K, Li Q, Alber F, Zhou XJ. Hi-corrector: A fast, scalable and memory-efficient package for normalizing large-scale hi-c data. Bioinformatics. 2015;31:960–2.

39. Ay F, Bailey TL, Noble WS. Statistical confidence estimation for hi-c data reveals regulatory chromatin contacts. Genome Res. 2014;24:999–1011.

40. Rao SS, Huntley MH, Durand NC, Stamenova EK, Bochkov ID, Robinson JT, et al. A 3D map of the human genome at kilobase resolution reveals principles of chromatin looping. Cell. 2014;159:1665–80.

41. Vidal E, Dily F le, Quilez J, Stadhouders R, Cuartero Y, Graf T, et al. OneD: Increasing reproducibility of hi-c samples with abnormal karyotypes. Nucleic Acids Res. 2018.
