# Supplementary figures and images for: SpectralTAD: an R package for defining a hierarchy of topologically associated domains using spectral clustering
Source: BMC Bioinformatics. 2020 Jul 20;21:319. doi: 10.1186/s12859-020-03652-w (PMC7372752; doi:10.1186/s12859-020-03652-w)

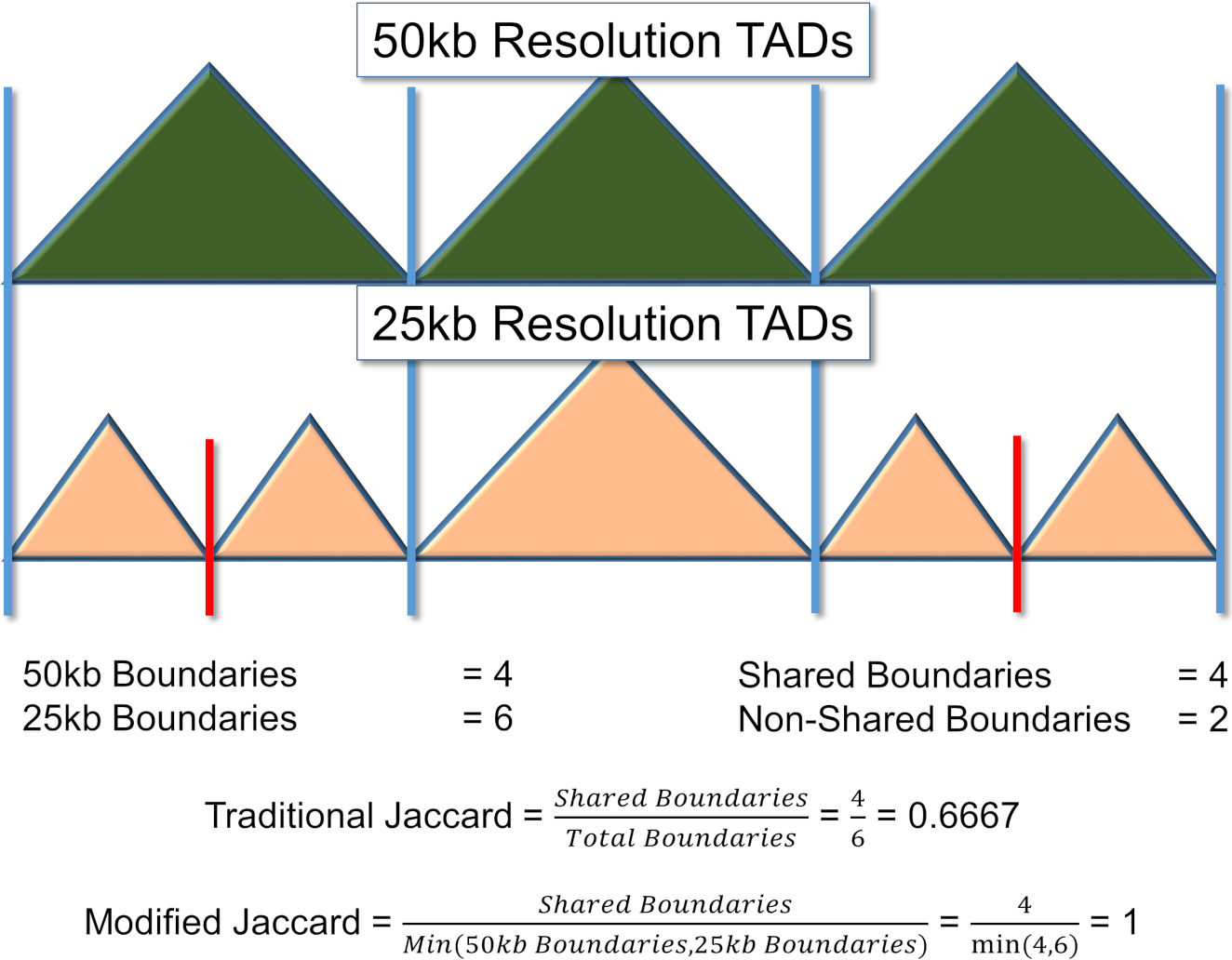

Supplement: Supplementary file 3 — Additional file 3: Figure S1. Example of modified Jaccard statistics to measure agreement between TAD boundaries detected at different resolutions. The top triangles indicate TADs detected at 50 kb resolution, while the bottom triangles indicate those detected at 25 kb resolution. There are four shared boundaries (blue lines) and two non-shared boundaries (red lines). The traditional Jaccard statistic underestimates the fact that the four TAD boundaries agree at a different resolution, while the modified Jaccard statistics correctly identifies the perfect overlap between TAD boundaries by ignoring resolution differences. [file 12859_2020_3652_MOESM3_ESM.tif]

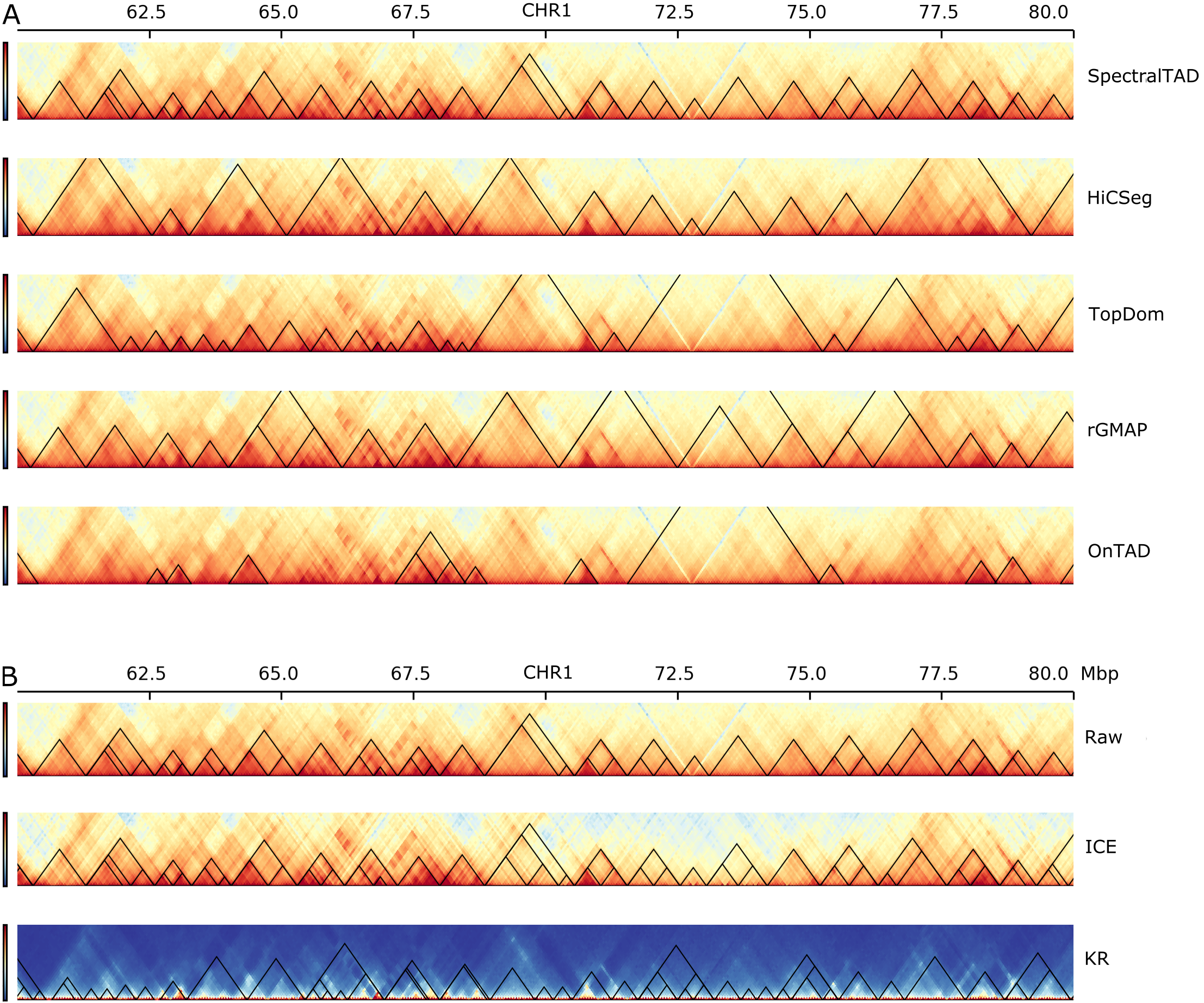

Supplement: Supplementary file 4 — Additional file 4: Figure S2. Examples of TADs detected under different conditions. A) TADs detected by SpectralTAD, TopDom, OnTAD, HiCSeg and rGMAP. B) TADs detected from raw, ICE-, and KR-normalized data. SqrtVC-normalized data could not be plotted due to format conversion issues. Red-yellow-blue color gradient indicate a high-medium low chromatin interaction strength; triangles indicate TADs. GM12878 data from [3], resolution 50 kb, chr1:60000000–80,000,000 (hg19). All parameters were set according to the instructions of each TAD caller. HiCExplorer v.3.0 [46] was used for visualization. [file 12859_2020_3652_MOESM4_ESM.png]

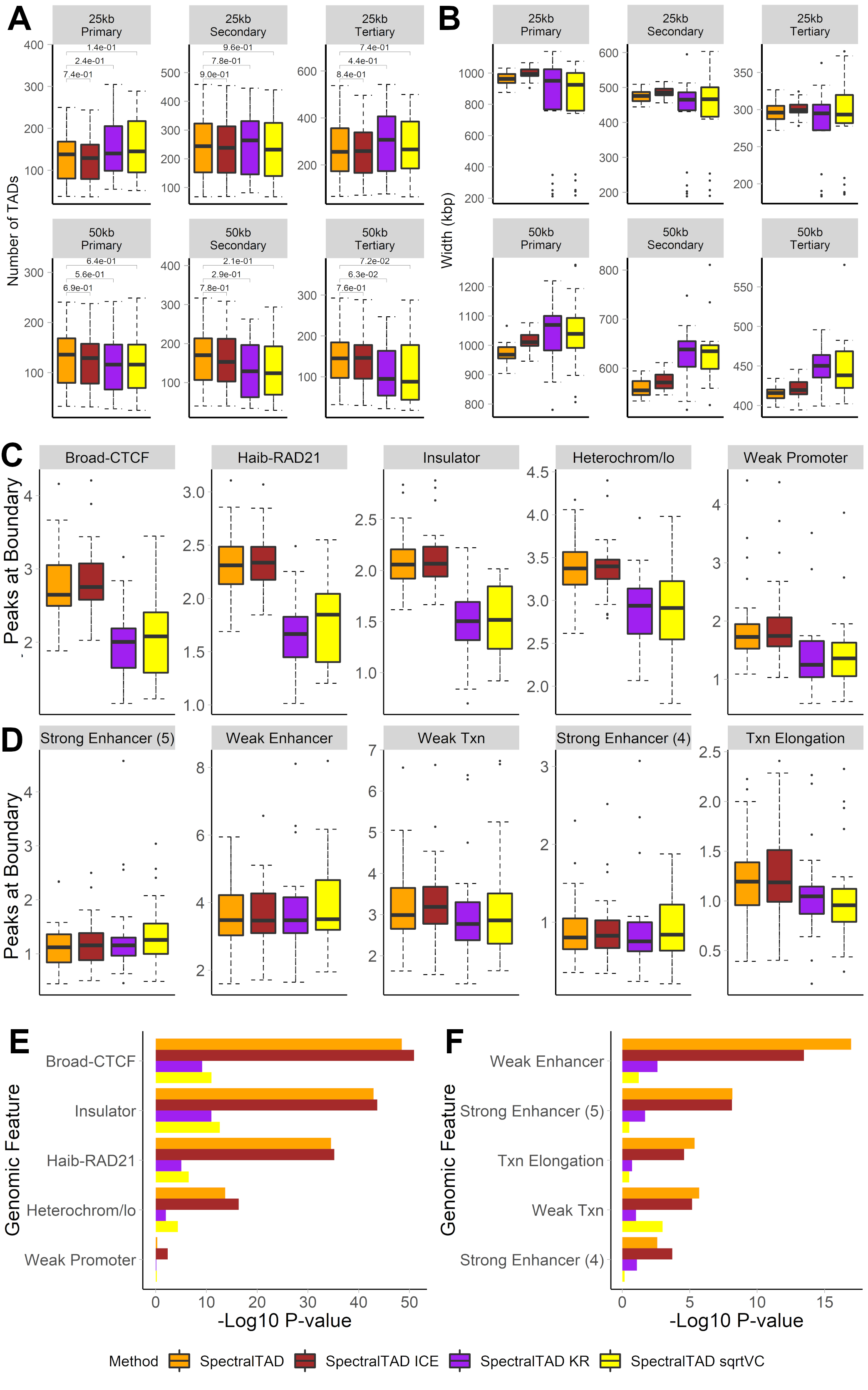

Supplement: Supplementary file 6 — Additional file 6: Figure S3. The effect of data normalization on the average number (A) and width in kilobases (B) of TADs and the average number of peaks in enriched markers (C) and depleted markers (D), enrichment (F) and depletion (G) for different genomic annotations. Counts (A) and widths (B) for raw, KR-, ICE- and sqrtVC-normalized GM12878 data at 25 kb and 50 kb resolutions, averaged across chromosome 1–22, are shown for primary, secondary, and tertiary TADs detected by SpectralTAD. The average number of annotations for enriched (D) and depleted (E) genomic features and the permutation p-values corresponding to enrichment (F), and depletion (G) for the top most enriched/depleted genomic annotations (permutation test) at TAD boundaries for GM12878 data at 50 kb resolution are shown. [file 12859_2020_3652_MOESM6_ESM.tif]

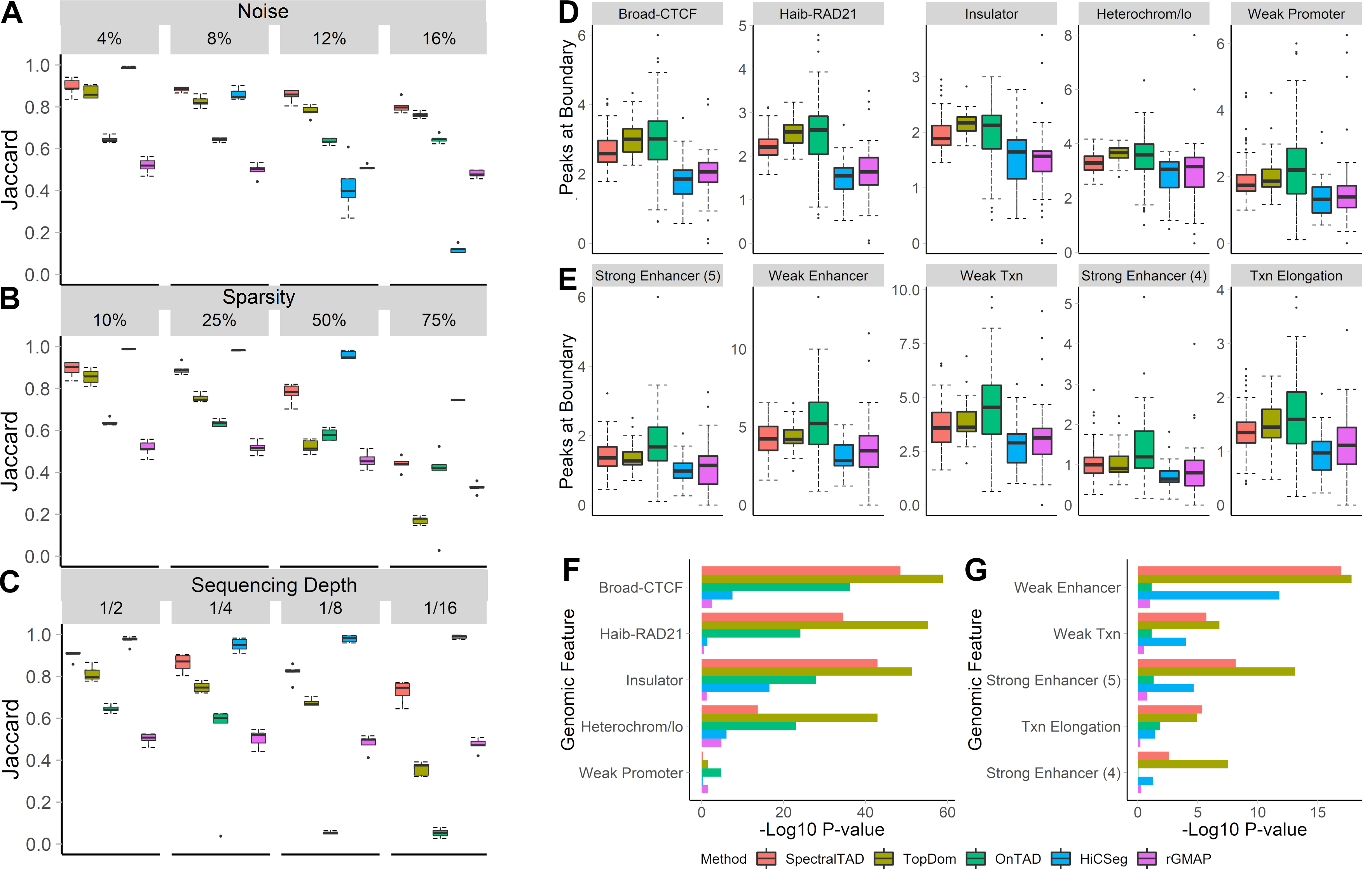

Supplement: Supplementary file 7 — Additional file 7: Figure S4. The comparison of SpectralTAD and other TAD callers regarding TAD consistency and biological significance. To test for robustness to noise, sparsity, and downsampling, TADs were called from simulated Hi-C matrices using SpectralTAD and other TAD callers. The TAD boundaries were extended by 50 kb regions flanking a boundary on both sides. They were compared with the ground-truth TADs using the Jaccard similarity metric. The performance of the TAD callers was assessed at a different level of noise (A, the percentage of the original contact matrix modified by adding a constant of two), sparsity (B, the percentage of the original contact matrix replaced with zero), and downsampling (C, the fraction of contacts kept, see Methods). Using the raw data from GM12878 at 50 kb resolution, enrichment of genomic annotations within 50 kb regions flanking a TAD boundary on both sides was assessed using a permutation test. The average number of annotations for enriched (D) and depleted (E) genomic features and the permutation p-values corresponding to enrichment (F), and depletion (G) for the top five most enriched/depleted genomic annotations are shown. Results averaged across chromosome 1–22 are shown. [file 12859_2020_3652_MOESM7_ESM.tif]

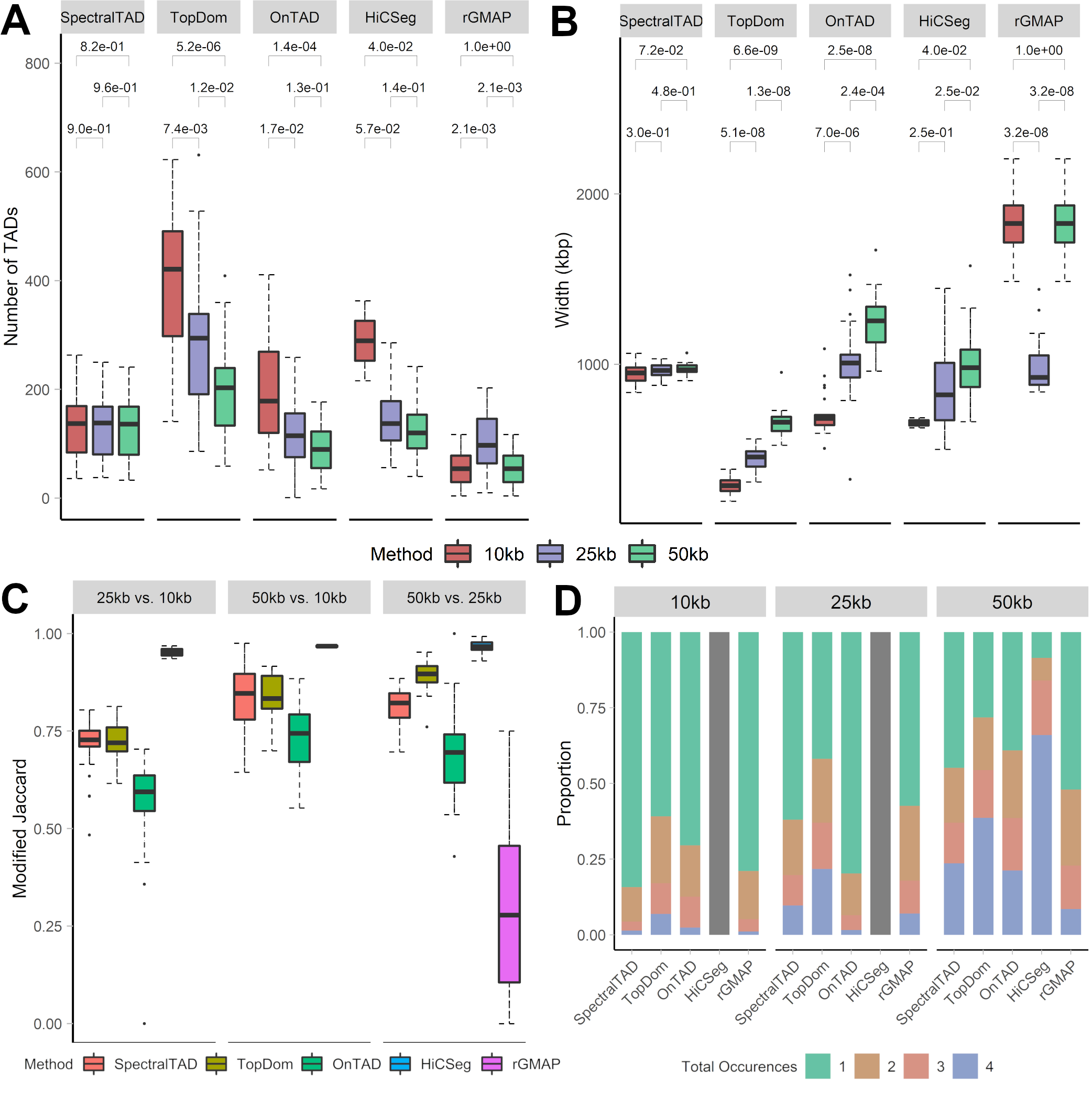

Supplement: Supplementary file 9 — Additional file 9: Figure S5. The number, width, and consistency of TADs called across resolutions and primary vs. replicate for different methods. The average number (A) and width (B) of TADs across resolutions, Jaccard similarity between TAD boundaries detected from primary and replicate data and modified Jaccard similarity between TAD boundaries detected from data at 10 kb, 25 kb and 50 kb resolutions (C) and the proportion of shared boundaries across four replicates (D) are shown. HiCseg failed to run on some data due to sparsity, as indicated by gray bars on panel D. Wilcoxon test p-values are shown. Data from the GM12878 cell line, chromosomes 1–22. [file 12859_2020_3652_MOESM9_ESM.tif]

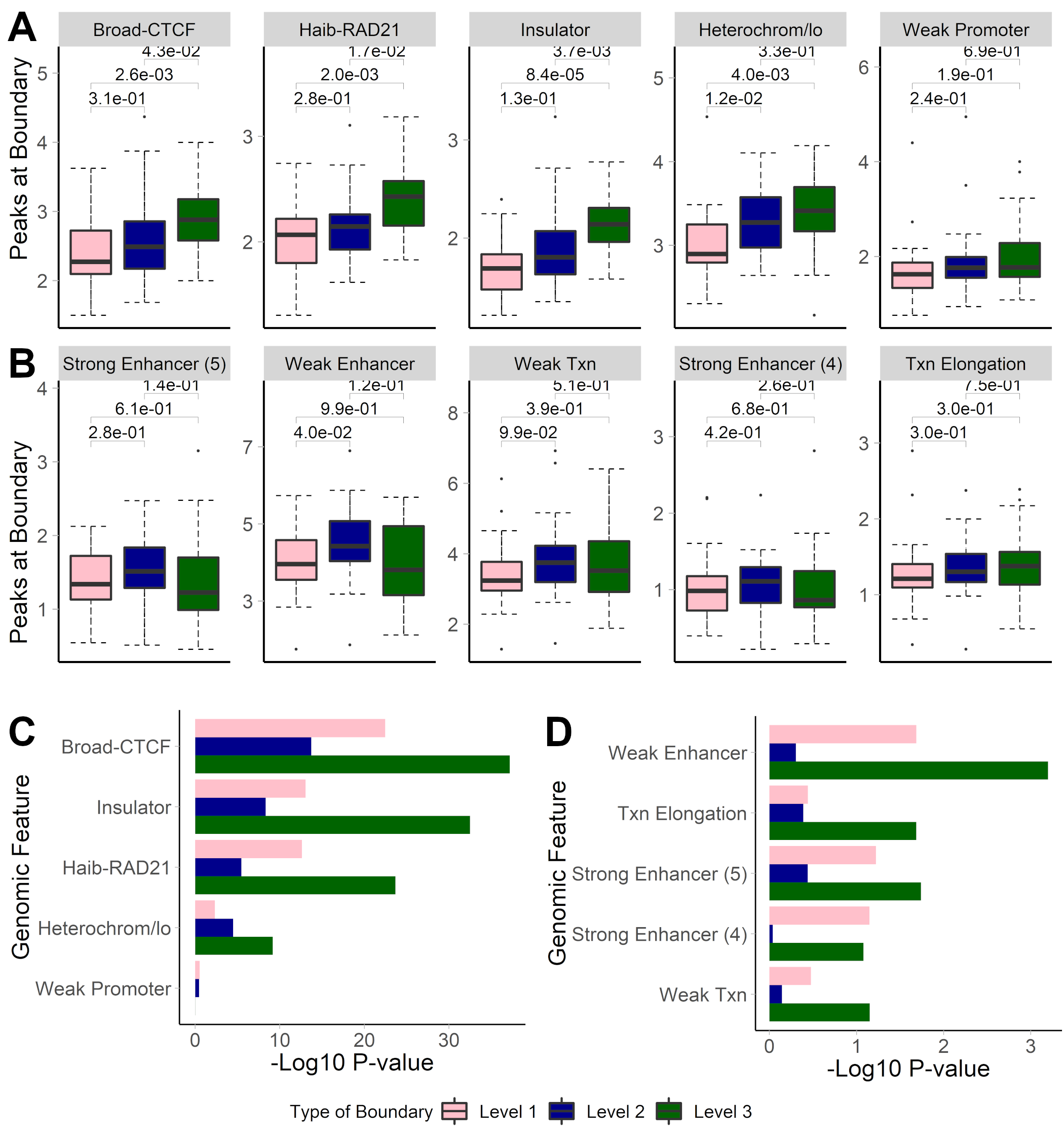

Supplement: Supplementary file 10 — Additional file 10: Figure S6. The effect of the hierarchy of TAD boundaries detected by SpectralTAD on the average number of annotations in enriched (A) and depleted (B) genomic markers and on enrichment (C) and depletion (D) for different genomic annotations. Results for TAD boundaries detected as Level 1, 2, and 3 boundaries are shown. Genomic annotations were considered within 50 kb regions flanking a boundary on both sides. Wilcoxon test p-values are shown in panel A & B, and aggregated p-values, using the Fisher’s method, are shown for panels C & D. Raw data from GM12878 cell line, chromosome 1–22, 50 kb resolution. [file 12859_2020_3652_MOESM10_ESM.tif]

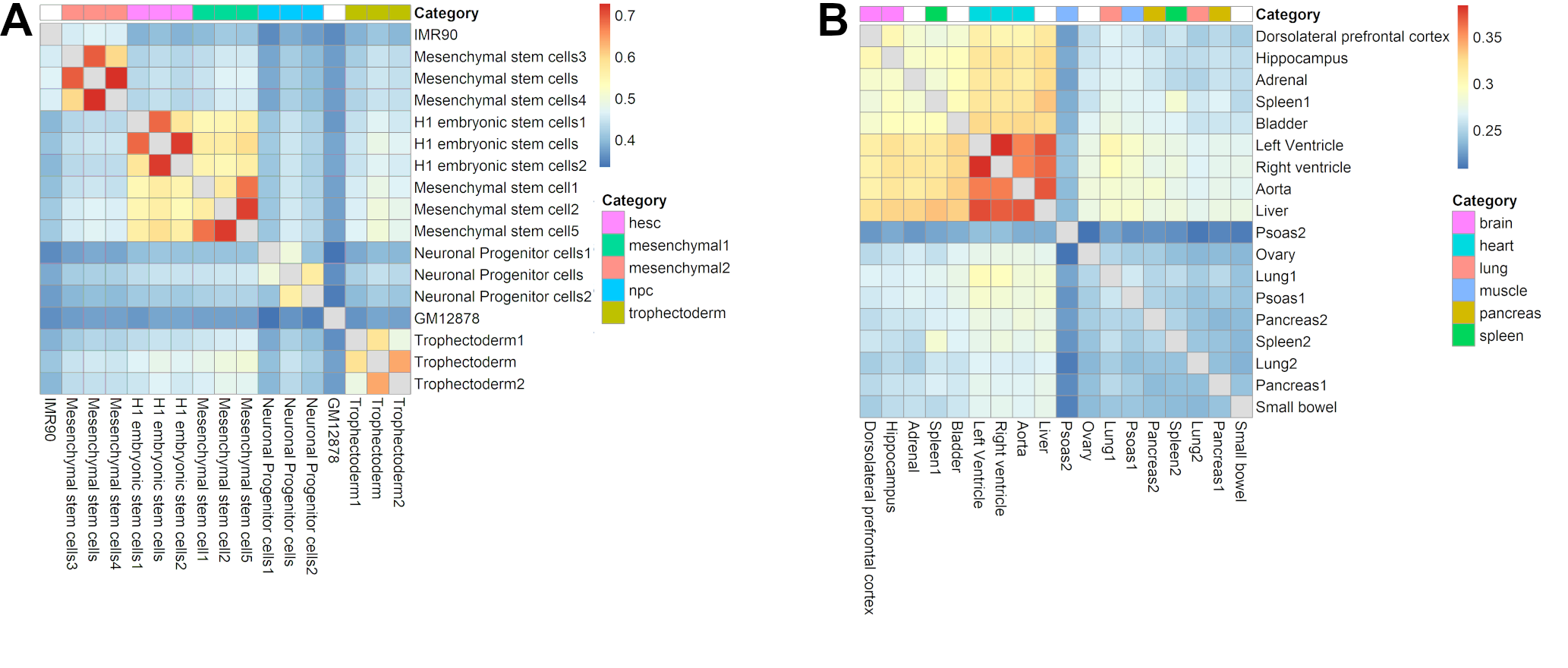

Supplement: Supplementary file 12 — Additional file 12: Figure S7. Jaccard similarity of TAD boundaries across cell types (A) and tissues (B). TADs were called using SpectralTAD. Clustering was performed using Ward clustering applied to a Jaccard distance matrix. All TADs were called on raw 40 kb data from [39]. Various cell-lines and tissues are used. [file 12859_2020_3652_MOESM12_ESM.tif]

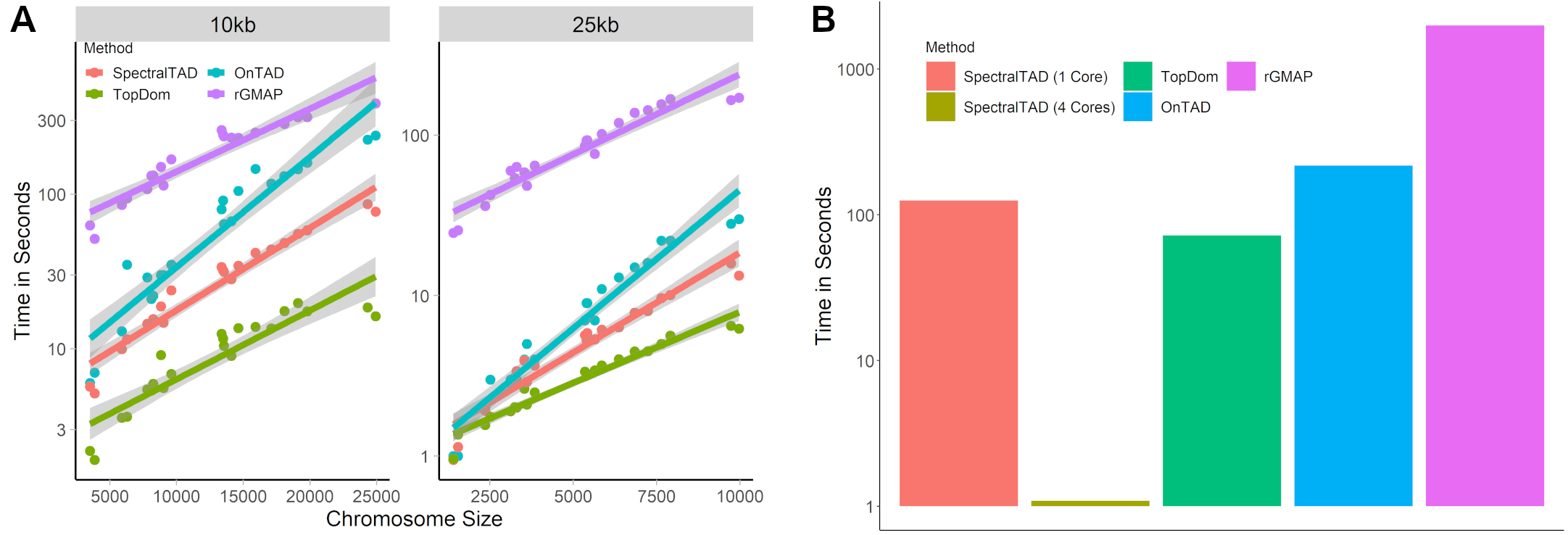

Supplement: Supplementary file 14 — Additional file 14: Figure S8. Runtime performance of various TAD callers. TADs were called using data from the GM12878 cell line at 10 kb and 25 kb resolution, and runtimes recorded. (A) Runtimes were summarized across different chromosomes. Each dot represents chromosome-specific run time averaged across three runs, with the regression line approximating the trend. X-axis – chromosome size in the number of bins, Y-axis – time in seconds. (B) The total time to analyze chromosomes 1–22 was calculated and summarized across methods and levels of parallelization for GM12878 25 kb resolution data. X-axis – Method, Y-axis – time in seconds. Results for HiCSeg are excluded due to exceptionally slow runtimes (24h hours for one 10 kb chromosome). [file 12859_2020_3652_MOESM14_ESM.tif]

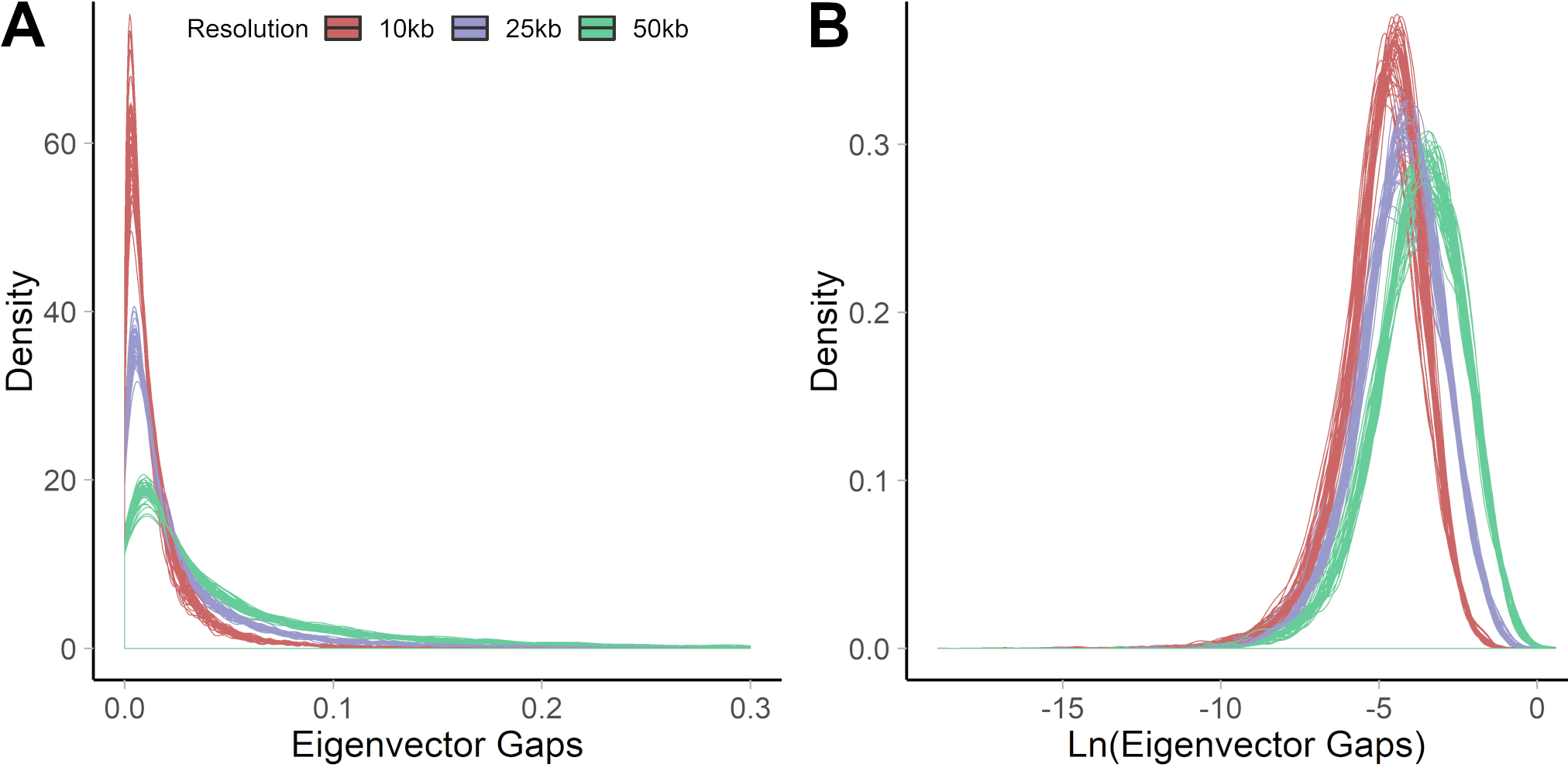

Supplement: Supplementary file 16 — Additional file 16: Figure S10. Distribution of eigenvector gaps. The distributions of eigenvector gaps are plotted separately for each 10 kb, 25 kb, and 50 kb contact matrix from [3], 131 chromosome-specific datasets total. Results are colored by resolution. Higher-resolution data shows smaller overall gaps due to a larger number of regions of high sparsity. The untransformed eigenvector gaps (A) and the natural log eigenvector gaps (B) are shown. MASS::fitdistr() function was used to establish the best fit by a lognormal (67 datasets) or a Weibull (64 datasets) distributions with similar log-likelihoods. The lognormal fit was chosen to model the distribution of log eigenvector gaps. [file 12859_2020_3652_MOESM16_ESM.tif]
