# Supplementary material for: SpectralTAD: an R package for defining a hierarchy of topologically associated domains using spectral clustering
Source: BMC Bioinformatics. 2020 Jul 20;21:319. doi: 10.1186/s12859-020-03652-w (PMC7372752; doi:10.1186/s12859-020-03652-w)
Supplement: Supplementary file 13 — Additional file 13: Table S5. Jaccard similarity across TAD hierarchy. Results for the corresponding comparison of Primary, Secondary, Tertiary TADs, and Level 1, 2, 3 TAD boundaries are shown. Jaccard similarity coefficients were compared using a Wilcoxon signed-rank test. Column p-values correspond to the comparison of Jaccard within levels between tissue samples and cell lines. Row p-values correspond to the comparisons within each type of data across the hierarchy. [file 12859_2020_3652_MOESM13_ESM.pdf]

	Primary vs. Secondary			Primary vs. Tertiary			Secondary vs. Tertiary		
	Primary	Secondary	P-value	Primary	Tertiary	P-value	Secondary	Tertiary	P-value
Cell Line	0.42	0.40	0.0001	0.42	0.35	<0.0001	0.40	0.35	<0.0001
Tissue	0.22	0.21	0.0003	0.22	0.18	<0.0001	0.21	0.18	<0.0001
P-value	<0.0001	<0.0001		<0.0001	<0.0001		<0.0001	<0.0001	
	Level 1 vs. Level 2			Level 1 vs. Level 3			Level 2 vs. Level 3		
	Level 1	Level 2	P-value	Level 1	Level 3	P-value	Level 2	Level 3	P-value
Cell Line	0.23	0.23	0.0347	0.23	0.30	<0.0001	0.23	0.30	<0.0001
Tissue	0.12	0.10	<0.0001	0.12	0.13	0.0600	0.10	0.13	<0.0001
P-value	<0.0001	<0.0001		<0.0001	<0.0001		<0.0001	<0.0001	
